# Supplementary material for: Protective efficacy of a SARS-CoV-2 DNA vaccine in wild-type and immunosuppressed Syrian hamsters
Source: NPJ Vaccines. 2021 Jan 25;6:16. doi: 10.1038/s41541-020-00279-z (PMC7835356; doi:10.1038/s41541-020-00279-z)
Supplement: Supplementary file 1 — Supplementary Information [file 41541_2020_279_MOESM1_ESM.pdf]

Supplementary Figure 1

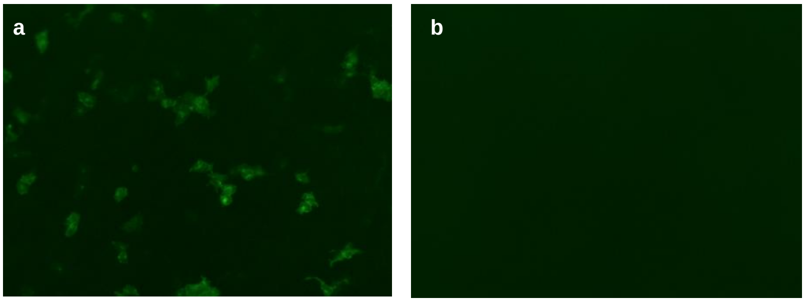

Supplementary Figure 1. Expression of pWRG/nCoV-S(opt) plasmid in 293T cells. Expression of the spike protein was confirmed by transfection of 293T cells followed by immunofluroesence antibody test (IFAT) using **a)** human convalescent plasma and compared to **b)** empty vector.

Supplementary Figure 2

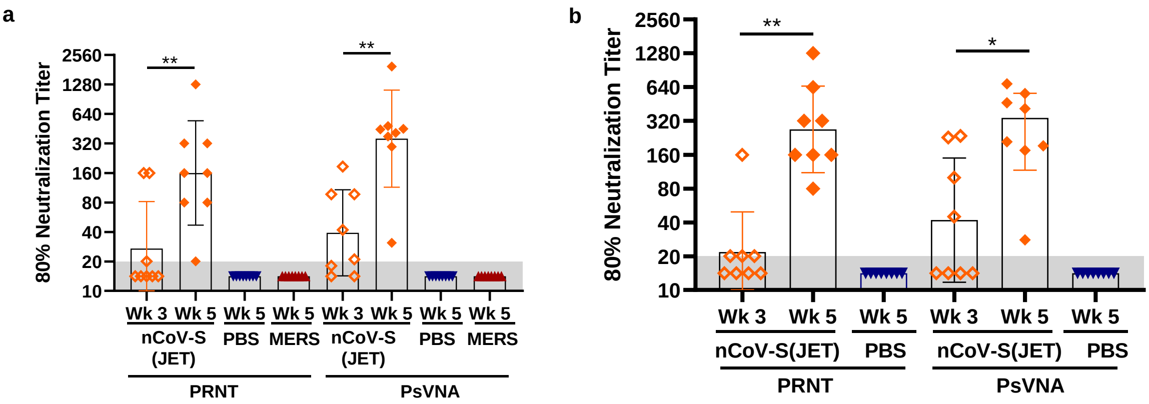

Supplementary Figure 2. Neutralization titers of hamsters vaccinated with nCoV-S(JET) DNA vaccine. Eighty percent neutralization titers by PRNT and PsVNA of hamsters shown in **Figs. 1b** and **2b** are plotted.

Supplementary Figure 3

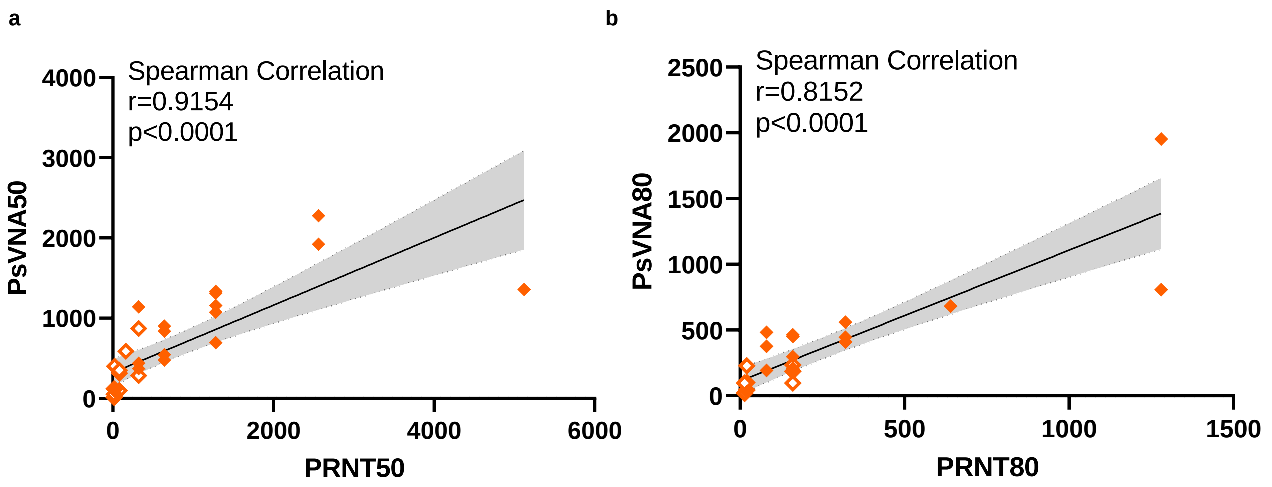

Supplementary Figure 3. Correlation of PRNT and PsVNA assays. a) Fifty and **b)** 80 percent neutralization titers by PRNT and PsVNA from hamsters vaccinated once (open symbols) or twice (closed symbols) with the nCoV-S(JET) vaccine from **Fig. 1** and **Fig. 2** are plotted (positive by at least one assay only). Correlation was analyzed by Spearman with an **a)** r=0.9154 and *P*<0.0001 and **b)** r=0.8152 and *P*<0.0001 with linear regression (black line) and 95% confidence intervals (shaded area) shown.

Supplementary Figure 4

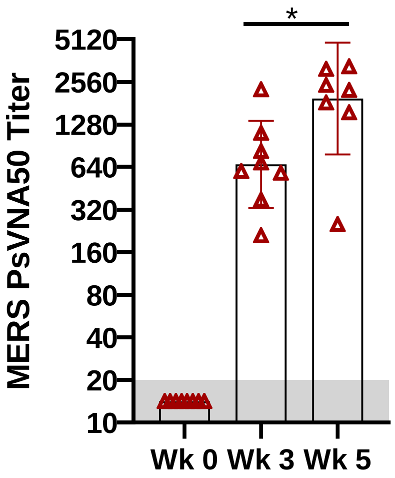

Supplementary Figure 4. Neutralization titers of hamsters vaccinated with MERS-CoV DNA vaccine. Eight hamsters were vaccinated with MERS-CoV DNA vaccine using Tropis. Serum collected prior to and after 1 and 2 vaccinations was analyzed by MERS PsVNA (LLOQ = 20, grey shade). Asterisks indicate that results were statistically significant, as follows: *, *P*<0.05.

Supplementary Figure 5

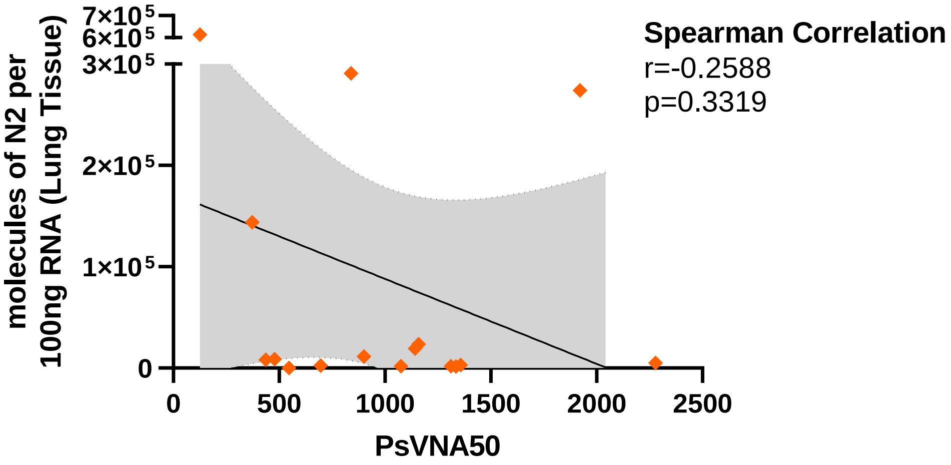

Supplementary Figure 5. Correlation of PsVNA50 titers and lung viral RNA. Fifty percent neutralization titers by PsVNA from hamsters vaccinated twice with nCoV-S(JET) vaccine are plotted against viral RNA detected in lung homogenates at the time of euthanasia (both **Fig. 1** and **Fig. 2**). Correlation was analyzed by Spearman with an r=-0.2588 and *P*=0.3319 with linear regression (black line) and 95% confidence intervals (shaded area) shown.
